# Modulation of Microstructure, Magnetic, and Magnetocaloric Properties in La_0.80_Ag_0.20_MnO_3_ via Eu/Pb Co-Doping

**DOI:** 10.3390/ma19091755

**Published:** 2026-04-25

**Authors:** Fucheng Zhu, Yang Xu, Yanghui Chu, Zekai Wang, Xingyu Hong, Huiyan Zhang, Hailing Li, Weihua Gu, Zhiyuan Liu, Juan Liu, Ailin Xia

**Affiliations:** 1 School of Materials Science and Engineering, Anhui University of Technology, Ma’anshan 243002, China; 15156923568@163.com (F.Z.); 17756545925@163.com (Y.X.); 17778758156@163.com (Y.C.); wzk01212@163.com (Z.W.); 19810764365@163.com (X.H.); lhl_nano@163.com (H.L.); guweihua0126@ahut.edu.cn (W.G.); zhiyuanliu826@ahut.edu.cn (Z.L.); jliu0922@ahut.edu.cn (J.L.); alxia@126.com (A.X.); 2 Advanced Ceramics Research Center, School of Materials Science and Engineering, Anhui University of Technology, Ma’anshan 243002, China

**Keywords:** La-based perovskite manganite, A-site doping, magnetocaloric effect

## Abstract

**Highlights:**

- Eu^3+^ replacing La^3+^ shirinks lattice, lengthening 〈*d*_Mn-O_〉 and lowering 〈*θ*_Mn-O-Mn_〉.
- Whereas the replacement of Pb^2+^ for Ag^+^ induces the opposite effect.
- Eu doping suppresses crystallization ability, while Eu/Pb co-doping avoids this.
- Eu doping lowers *T*_C_ by weakened DE, Pb doping raises *T*_C_ by enhanced DE.
- Co-doping optimizes *T*_C_ (299.3 K) and achieves highest |Δ*S*_M_^max^| (3.90 J·kg^−1^·K^−1^).

**Abstract:**

Four perovskite manganite samples, La_0.80_Ag_0.20_MnO_3_ (LA), La_0.78_Eu_0.02_Ag_0.20_MnO_3_ (LEA), La_0.80_Pb_0.05_Ag_0.15_MnO_3_ (LPA), and La_0.77_Eu_0.03_Pb_0.05_Ag_0.15_MnO_3_ (LEPA), were prepared by the Pechini sol–gel method. The samples were characterized by X-ray diffraction, scanning electron microscopy, energy-dispersive spectroscopy, X-ray photoelectron spectroscopy, and a magnetic property measurement system. A systematic investigation was conducted into the individual effects of Eu and Pb doping, as well as their co-doping, on the microstructural, magnetic and magnetocaloric properties of the materials. The results show that all samples are mainly composed of a rhombohedral perovskite phase with the $R\bar{3}c$ space group, accompanied by a trace amount of Ag. Addition of Eu^3+^ and Pb^2+^ induces lattice contraction and expansion, respectively. Under the same processing conditions, the average crystallite and particle sizes of the LEA sample (45.3 nm and 0.18 μm) are smaller than those of the other three samples (69.6~80.6 nm and 0.38~0.44 μm), indicating that the introduction of Eu alone suppresses crystallization ability, which can be avoided through Eu/Pb co-doping. All samples undergo a second-order ferromagnetic–paramagnetic transition, and the Curie temperature *T*_C_ shifts to either lower or higher temperatures upon the introduction of Eu or Pb alone (from 310.8 K to 298.0 K or 318.0 K, respectively), which is attributed to the variation of the Mn^3+^/Mn^4+^ double-exchange (DE) interaction resulting from the ionic size mismatch and lattice distortion. In the LPA sample, an additional contribution arises from the altered Mn^3+^/Mn^4+^ ratio and enhanced DE interaction caused by the substitution of Pb^2+^ for Ag^+^. By modifying the Eu/Pb ratio, the *T*_C_ of the LEPA sample was tuned to 299.3 K, and its maximum magnetic entropy change was enhanced to 3.90 J·kg^−1^·K^−1^ (∆*H* = 2 T). These results indicate that multicomponent synergistic regulation can improve the magnetocaloric performance of La-based perovskite manganites, providing a useful strategy for the development of room-temperature magnetic refrigeration materials.

## 1. Introduction

As global energy consumption continues to rise and natural resources become increasingly depleted, magnetocaloric refrigeration, driven by the magnetocaloric effect (MCE), has attracted considerable interest as a promising alternative to conventional vapor compression technology. Its advantages include zero greenhouse gas emissions, high efficiency (20–50% higher than that of conventional vapor compression), and a compact system design (with solid refrigerant) [1]. Magnetic refrigeration technology has been practically applied in the low-temperature regime, particularly in the successful use of adiabatic demagnetization refrigerators in the sub-Kelvin region (<1 K) for scenarios such as space science missions and low-temperature physics laboratory research [2]. However, room-temperature magnetic refrigeration still faces several challenges, primarily related to material properties, heat transfer efficiency, magnet design and system cost, among other factors [3].

Gd metal serves as the benchmark material for room-temperature magnetic refrigerators. It has a Curie temperature *T*_C_ of about 293 K and achieves a specific entropy change of 13.22 J·kg^−1^·K^−1^ under a 7 T magnetic field. However, Gd suffers from poor corrosion resistance and a limited operating temperature range [4]. Researchers have been dedicated to developing more cost-effective magnetocaloric materials since the discovery of the giant magnetocaloric effect in Gd_5_(Ge_1−*x*_Si*_x_*)_4_ compounds in the late 1990s. So far, three classes of materials have emerged as potential alternatives to Gd-based alloys: R_1−*x*_A*_x_*MnO_3_ manganites, La(Fe, Mn, Co, Si)_13−*x*_Si*_x_*H_y_ hydrides, and MnFe(P, As, Si, Ge) compounds [5]. Among these, first-order phase transition materials (e.g., La-Fe-Si-based and Mn-Fe-P-Ge-based alloys) exhibit excellent magnetocaloric performance in laboratory settings but suffer from pronounced magnetic and thermal hysteresis, as well as insufficient mechanical stability for practical device applications [3]. By contrast, perovskite materials, especially the manganites (La,R)_1−*x*_M*_x_*MnO_3_ (where R represents trivalent rare earth ions and M stands for divalent or monovalent cations), have become ideal substitutes for Gd. They offer low-cost, facile preparation, tunable *T*_C_, good chemical stability, small thermal hysteresis, and superior magnetocaloric effect [4]. Studies show that La_2/3_(Ca,Sr)_1/3_MnO_3_ achieves a cooling power of 35 W·kg^−1^ at room temperature, much higher than the 16 W·kg^−1^ of Gd plates under the same conditions [6].

A-site doping is an effective method for tuning the magnetic and magnetocaloric properties of manganites. Doping with trivalent ions primarily alters lattice distortion, while divalent or monovalent doping can further adjust the Mn valence state (Mn^3+^/Mn^4+^). Through these effects, A-site doping regulates the double-exchange (DE) interaction between Mn^3+^-O^2−^-Mn^4+^ and modulates spin–lattice coupling, thereby influencing the magnetic transition and magnetocaloric response [7]. For instance, in La_0.7−*x*_Eu*_x_*Sr_0.3_MnO_3_, increasing Eu^3+^ content decreases *T*_C_ from 357 to 228 K, while maximum magnetic entropy change ($\left|∆S_{M}^{\max}\right|$) increases from 3.43 to 4.55 J·kg^−1^·K^−1^ under the field change of 5 T. This behavior is mainly attributed to the Eu^3+^-substitution-induced reduction in A-site ionic radius, which increases lattice distortion and decreases the Mn-O-Mn bond angle, thereby weakening the DE interaction and lowering *T*_C_, while enhancing spin–lattice coupling to improve the magnetic entropy change and refrigeration capacity [8]. Bouzid et al. found that Pb^2+^ substitution shifts the magnetic transition toward a higher temperature and simultaneously improves the magnetocaloric response; in La_0.9_K_0.1_MnO_3_ and La_0.8_K_0.1_Pb_0.1_MnO_3_, *T*_C_ increases from 178 to 289 K, while $\left|∆S_{M}^{\max}\right|$ increases from 3.04 to 5.5 J·kg^−1^·K^−1^ under 5 T. The co-substitution of Pb^2+^ and K^+^ for La^3+^ optimizes the Mn^3+^/Mn^4+^ ratio for the DE interaction, thus increasing both *T*_C_ and saturation magnetization. Meanwhile, Pb doping promotes grain growth and densification, optimizing the magnetic ordering network. Consequently, the La_0.8_K_0.1_Pb_0.1_MnO_3_ exhibits a second-order phase transition with the *T*_C_ close to room temperature (289 K) and enhanced magnetocaloric performance [9].

Among perovskite manganites for near-room-temperature magnetic refrigeration, La_1−*x*_Ag*_x_*MnO_3_ features a tunable *T*_C_ and favorable magnetocaloric performance [10,11]. More specifically, La_0.80_Ag_0.20_MnO_3_ shows a *T*_C_ of 308 K and $\left|∆S_{M}^{\max}\right|$ of 3.20 J·kg^−1^·K^−1^ under a field change of 2 T [12]. In the present study, this composition was used as the base material, and La_0.80_Ag_0.20_MnO_3_, La_0.78_Eu_0.02_Ag_0.20_MnO_3_, La_0.80_Pb_0.05_Ag_0.15_MnO_3_ and La_0.77_Eu_0.03_Pb_0.05_Ag_0.15_MnO_3_ samples were prepared using the sol–gel method. The individual and combined effects of Eu and Pb doping on the microstructural, magnetic, and magnetocaloric properties of the materials were systematically investigated. The results indicate that, compared with individual Eu^3+^ or Pb^2+^ doping, Eu^3+^/Pb^2+^ co-doping enhances the magnetocaloric effect while bringing *T*_C_ closer to room temperature, providing a reference for the performance study of magnetocaloric materials.

## 2. Materials and Methods

The La_0.80_Ag_0.20_MnO_3_ (LA), La_0.78_Eu_0.02_Ag_0.20_MnO_3_ (LEA), La_0.80_Pb_0.05_Ag_0.15_MnO_3_ (LPA) and La_0.77_Eu_0.03_Pb_0.05_Ag_0.15_MnO_3_ (LEPA) manganites were synthesized using the Pechini sol–gel method. Stoichiometric amounts of La(NO_3_)_3_·6H_2_O (Ourchem, Guangzhou, China, 99.0%), Eu(NO_3_)_3_·6H_2_O (Ourchem, 99.0%), Pb(NO_3_)_2_ (SCR, 99.0%), AgNO_3_ (SCR, 99.8%), and Mn(NO_3_)_2_·4H_2_O (Ourchem, 97.5%) were dissolved in 30 mL of distilled water under continuous stirring to form a homogeneous aqueous solution. Subsequently, appropriate amounts of citric acid and ethylene glycol were added as chelating agents. The resulting solution was maintained in a water bath at 100 °C for 2–3 h, until a yellow viscous polymeric gel (resin) was formed, and then cooled to room temperature for 30 min. To ensure complete removal of residual organic species, the resin was further heated at 300 °C for 30 min. The obtained precursor was then ground thoroughly and calcined at 1100 °C for 6 h to yield the final perovskite manganite powders.

The phase structure of the samples was analyzed using X-ray diffraction (XRD, Rigaku Ultima IV, Rigaku Corporation, Tokyo, Japan) with Cu-Kα radiation (λ = 1.5406 Å) over a 2*θ* range of 20–80°. Crystallographic parameters were refined using the Rietveld method with the FULLPROF program [13]. The microstructure and elemental composition were characterized using scanning electron microscopy (SEM, FEI NANO SEM430, FEI Company, Hillsboro, OR, USA) coupled with energy-dispersive X-ray spectroscopy (EDX). The elemental chemical states and surface compositions were examined using X-ray photoelectron spectroscopy (XPS, Nexsa G2, Thermo Fisher Scientific, Waltham, MA, USA) with Al Kα radiation. Magnetic measurements were carried out with a superconducting quantum interference device (SQUID) integrated into a magnetic property measurement system (MPMS, Quantum Design MPMS3, Quantum Design, San Diego, CA, USA). Temperature-dependent dc magnetization (*M*-*T*) curves were recorded during the heating process after zero-field cooling (ZFC) under an applied field of 0.05 T. Isothermal magnetization (*M*-*H*) curves were measured in the field range of 0–2 T at selected temperatures, with the temperature interval of 1 K and 2 K in vicinity of and far away from the Curie temperature.

## 3. Results and Discussion

**Figure 1:**
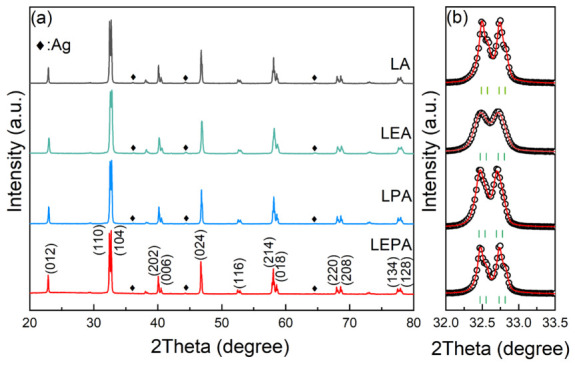
(**a**) XRD patterns of the LA, LEA, LPA, and LEPA samples and (**b**) the zoom of XRD patterns between 32 and 33.5 degree (black circles) with Rietveld profile fitting (red lines). The vertical solid lines indicate the Bragg-reflection positions corresponding to $R\bar{3}c$ phase.

**Figure 2:**
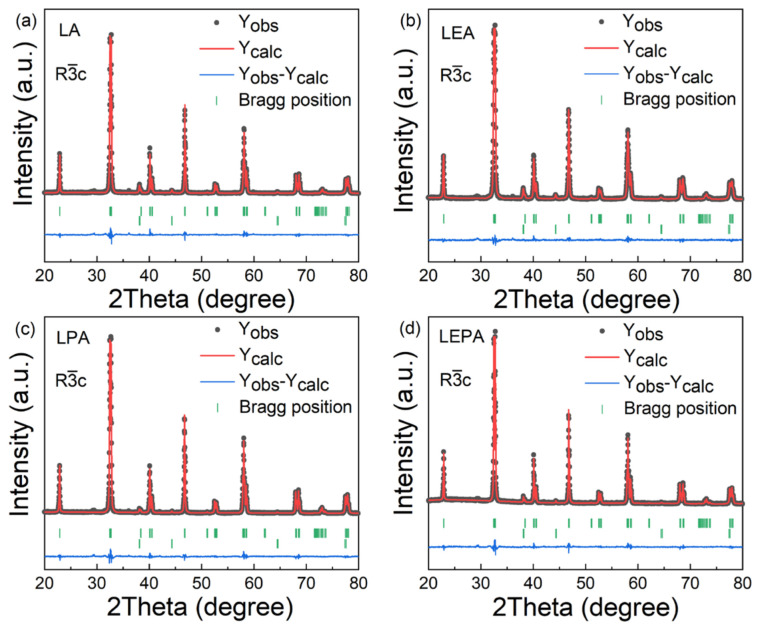
XRD Rietveld refinement patterns of the (**a**) LA, (**b**) LEA, (**c**) LPA, and (**d**) LEPA samples.

Figure 1 and Figure 2 present the X-ray diffraction (XRD) patterns of the samples and their Rietveld refinement results, respectively. All samples were identified as having a rhombohedral perovskite structure with the space group $R\bar{3}c$, together with a small amount of metallic Ag impurity phase, which is attributed to the very limited solubility of Ag in LaMnO_3_ [14]. Structural parameters, including the lattice constants (*a*, *b*, *c*), unit cell volume (*V*), Mn-O bond length (〈$d_{\mathrm{Mn}\text{-}O}$〉), and Mn-O-Mn bond angle (〈$\theta_{\mathrm{Mn}\text{-}O\text{-}\mathrm{Mn}}$〉), were obtained by Rietveld refinement of the XRD data. The relevant results are summarized in Table 1. Substitution of smaller Eu^3+^ for La^3+^ tends to cause lattice contraction, which enhances MnO_6_ octahedral tilting [15], leading to an increase in 〈$d_{\mathrm{Mn}\text{-}O}$〉 and a decrease in 〈$\theta_{\mathrm{Mn}\text{-}O\text{-}\mathrm{Mn}}$〉, whereas the replacement of larger Pb^2+^ for Ag^+^ induces the opposite effect, i.e., lattice expansion, shortening of Mn-O bonds and increasing Mn-O-Mn bond angles.

The stability of the perovskite structure is determined by the Goldschmidt tolerance factor 〈*t*〉, which reflects the degree to which the crystal structure deviates from the ideal cubic perovskite structure. The 〈*t*〉 value is calculated using the following equation [16]:

(1) $$\langle t\rangle \;=\;\frac{\left(\langle r_{A}\rangle \;+\;r_{O}\right)}{\sqrt{2}\left(r_{B}\;+\;r_{O}\right)}$$

where 〈*r*_A_〉, *r*_B_, and *r*_O_ represent the average ionic radii of the A, B, and O sites in perovskite manganites, respectively. In this study, the following ionic radii were used [17,18,19]: for A-site cations (CN = 12), $r_{{\mathrm{La}}^{3+}}$ = 1.36 Å, $r_{{\mathrm{Eu}}^{3+}}$ = 1.226 Å, $r_{{\mathrm{Pb}}^{2+}}$ = 1.49 Å, $r_{{\mathrm{Ag}}^{+}}$ = 1.46 Å; for B-site cations (CN = 6), $r_{{\mathrm{Mn}}^{3+}}$ = 0.645 Å, $r_{{\mathrm{Mn}}^{4+}}$ = 0.53 Å; for O anions, $r_{O^{2-}}$ = 1.40 Å. According to Equation (1), the 〈*t*〉 values for the four samples range from 0.9797 to 0.9834, consistent with the 0.96 < *t* < 1 range, which indicates stable rhombohedral perovskite structures [1]. Moreover, Pb^2+^ doping leads to a larger deviation of *t* from 1 than Eu^3+^ doping, indicating a greater distortion from the ideal cubic geometry and thus a more pronounced average lattice distortion [20]. The variance of the A-site ionic radius, *σ*^2^, can be used to quantify the degree of lattice disorder caused by doping different metal ions at the A-site. Its calculation is as follows [21]:

(2) $$\sigma^{2}\;=\;\sum y_{i}r_{i}^{2}-{\langle r_{A}\rangle }^{2}$$

where *r*_i_ is the ionic radius of each A-site ion and *y*_i_ is its occupancy. In this system, Eu^3+^ doping with a higher *σ*^2^ value has a greater influence on the local deformation of the MnO_6_ octahedra than Pb^2+^ doping [22].

Given that 〈$d_{\mathrm{Mn}\text{-}O}$〉 and 〈$\theta_{\mathrm{Mn}\text{-}O\text{-}\mathrm{Mn}}$〉 determine the electron transfer pathway in manganites, the bandwidth (*W*) derived from these structural parameters reflects the degree of Mn_3d_-O_2p_ orbital overlap and the effective hopping capability of e_g_ electrons. *W* is given by the following equation [21]:

(3) $$W\;=\;\frac{cos[\frac{\left(\pi -\langle \theta_{Mn\text{-}O\text{-}Mn}\rangle \right)}{2}]}{{\left(\langle d_{Mn\text{-}O}\rangle \right)}^{3.5}}$$

As discussed above, Eu^3+^ and Pb^2+^ doping exert different influences on the 〈$d_{\mathrm{Mn}\text{-}O}$〉 and 〈$\theta_{\mathrm{Mn}\text{-}O\text{-}\mathrm{Mn}}$〉. Compared with the LA sample, LEA and LPA exhibit smaller and larger *W*, with the corresponding weaker and stronger Mn_3d_-O_2p_ orbital overlap and DE interaction, which usually results in lower and higher *T*_C_, respectively [16].

The Scherrer equation was used to estimate the average crystallite size (*D*_SC_) of the samples [23]:

(4) $$D_{\mathrm{SC}}\;=\;\frac{0.89\;\times \;\lambda }{\beta \;\times \cos\theta }$$

where *λ* is the X-ray wavelength, *β* is the full width at half maximum (FWHM) of the diffraction peak, and *θ* is the Bragg angle of the diffraction peak. The *D*_SC_ of the LA, LEA, LPA and LEPA samples were observed to be 74.3, 45.3, 69.6 and 80.6 nm, respectively. Scherrer’s method only considers particle size effect on XRD peak broadening, ignoring lattice microstructure. In contrast, the Williamson–Hall (W-H) method accounts for microstrain-induced peak broadening. The W-H method is given by Equation (5), and the average particle size *D*_W-H_ and microstrain *ε* are determined as follows [24]:

(5) $$\beta \cos\theta \;=\;\frac{K\lambda }{D_{W\text{-}H}}\;+\;4\varepsilon \sin\theta$$

**Figure 3:**
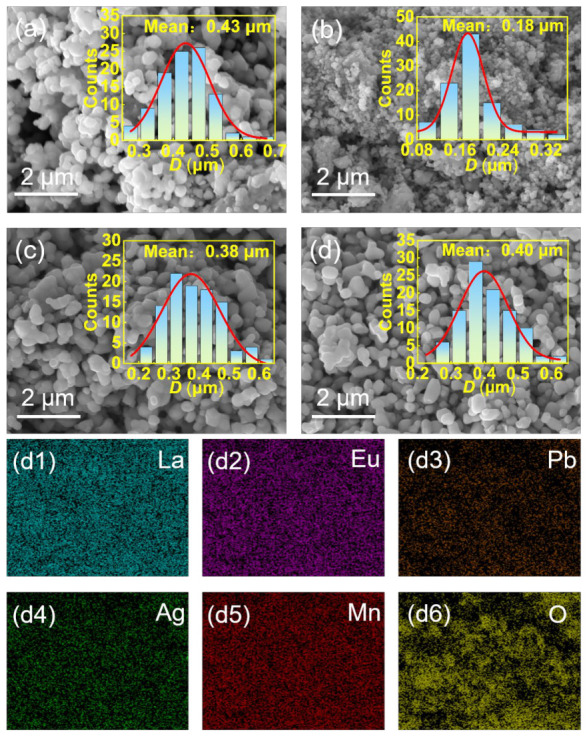
SEM images of the (**a**) LA; (**b**) LEA; (**c**) LPA; and (**d**) LEPA samples; The insets show the particle size distribution histograms and the red lines are the normal distribution fitting curves; (**d1**–**d6**) are the EDX elemental mapping of the LEPA sample.

The related results are summarized in Table 1. The crystallite sizes calculated by the W-H model are larger than the Scherrer-derived values because the strain contribution is eliminated, confirming the peak broadening arises from both small crystallite size and deformation. Among all samples, LEA exhibits the smallest *D*_W-H_, indicating that its reduced crystallite size is not merely due to strain broadening. The higher microstrain in LEA can be ascribed to its reduced crystallite size. As the crystallite size decreases, the ratio of surface atoms to volume atoms increases, generating more broken bonds on the crystallite surface and thereby enhancing the microstrain [25]. Figure 3 shows the scanning electron microscope (SEM) images of all the samples, with the EDX mapping of the LEPA sample (Figure 3(d1–d6)) as a representative. Each sample exhibits a morphology composed of nearly spherical and randomly dispersed particles. Gaussian fitting of the particle size histograms yields the average particle sizes (*D*_SEM_) of 0.44, 0.18, 0.38 and 0.40 μm for LA, LEA, LPA, and LEPA, respectively. The *D*_SEM_ is larger than the *D*_SC_ owing to each particle consisting of multiple crystallites. The agglomeration degree can be evaluated by the *D*_SEM_/*D*_W-H_ ratio [21], and the values for LA, LEA, LPA, and LEPA are 3.6, 2.4, 3.3, and 3.0, respectively. This ratio approximately represents the average number of crystallites in a single particle. The lower ratio of LEA implies a relatively lower degree of agglomeration than that of the other samples. Notably, the *D*_W-H_ and *D*_SEM_ of LEA are smaller than those of the other samples, suggesting that Eu^3+^ doping alone suppresses crystallization ability, whereas Eu/Pb co-doping avoids this issue.

Table 2 lists the actual compositions of all the samples determined by EDX analysis. The results confirm that Eu and Pb were incorporated into the samples, with their contents in good agreement with the nominal values. The lower Ag content is consistent with the presence of a small amount of metallic Ag phase observed in the XRD results.

**Figure 4:**
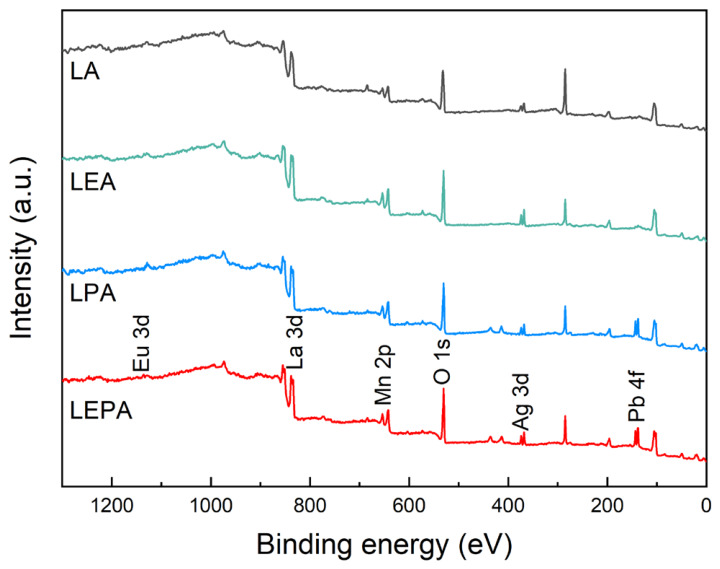
XPS survey spectra of the LA; LEA; LPA; and LEPA samples.

**Figure 5:**
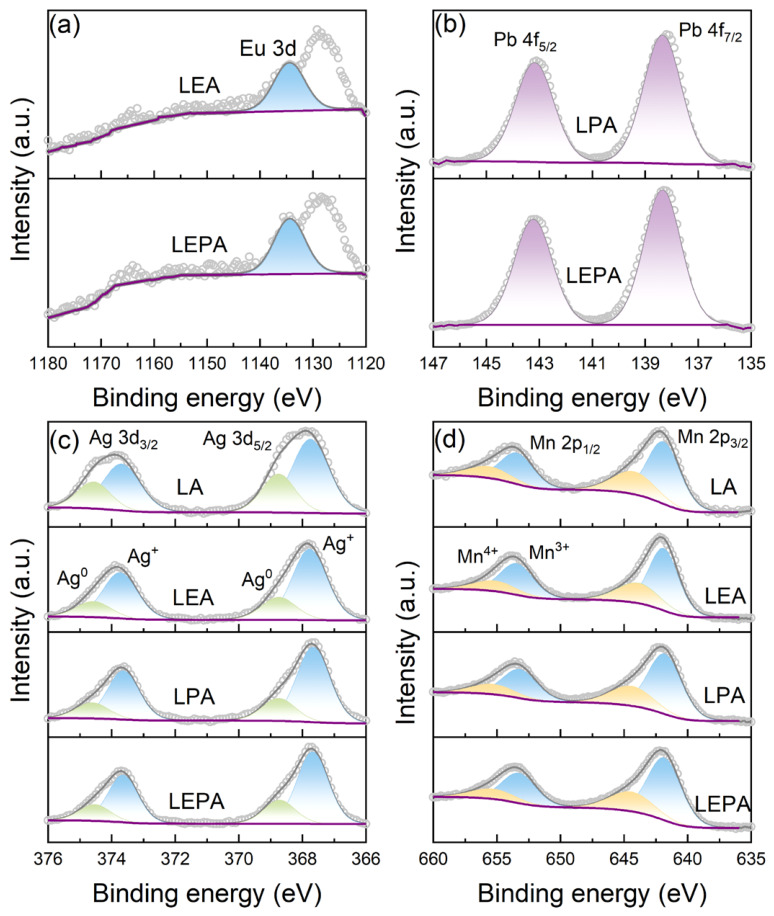
Experimental (light grey open circles) and deconvoluted (dark grey solid lines) high-resolution XPS spectra of the perovskite manganites in the present work: (**a**) Eu 3d, (**b**) Pb 4f, (**c**) Ag 3d and (**d**) Mn 2p.

X-ray photoelectron spectroscopy (XPS) was used to analyze the cationic chemical states and surface elemental composition of the perovskite manganite samples in the present work. Figure 4 displays the survey XPS spectra, where core levels of La 3d, Eu 3d, Pb 4f, Ag 3d, Mn 2p, and O 1s were detected on the sample surfaces. Figure 5a presents the characteristic peak of the Eu 3d orbital located at 1134.2 eV, indicating that the Eu element in LEA and LEPA mainly exists as Eu^3+^ [26]. Figure 5b reveals the high-resolution Pb 4f spectra of the LPA and LEPA samples, where two distinct peaks are observed at approximately 138.4 eV and 143.2 eV, corresponding to Pb 4f_7/2_ and Pb 4f_5/2_, with a spin–orbit splitting of about 4.8 eV. This result is consistent with the binding energy range reported for Pb^2+^ in the literature, suggesting that Pb primarily exists as Pb^2+^ [27]. The high-resolution Ag 3d spectrum (Figure 5c) features two main peaks at approximately 374.1 eV and 368.1 eV, assigned to Ag 3d_3/2_ and Ag 3d_5/2_. Peak fitting resolves two sets of sub-peaks, with higher binding energy peaks corresponding to metallic Ag (Ag^0^) and lower binding energy to Ag^+^ [28,29]. This result confirms the presence of both Ag^0^ and oxidized Ag, consistent with the XRD results, which detected a small amount of metallic Ag phase.

Figure 5d shows the high-resolution Mn 2p XPS spectra of the four samples with peak analysis results. Two main peaks appear at approximately 641.8 eV and 653.4 eV, corresponding to Mn 2p_3/2_ and Mn 2p_1/2_. The binding energies align well with literature ranges for Mn^3+^ and Mn^4+^, indicating a mixed valence state [30]. The coexistence of Mn^3+^ and Mn^4+^ is a crucial prerequisite for the DE interaction, where magnetism depends on electron transfer from Mn^3+^ ions ($t_{2g}^{3}e_{g}^{1}$) to adjacent Mn^4+^ ions ($t_{2g}^{3}e_{g}^{0}$) [31]. From these spectra, the Mn^3+^/Mn^4+^ molar ratio and Mn^3+^ content for each sample were calculated and are summarized in Table 3. The experimentally obtained Mn^3+^ ratio is generally higher than the theoretical value. This deviation, combined with the XRD detection of metallic Ag and the Ag 3d XPS results, is primarily due to the actual Ag content being lower than the theoretical value. Additionally, partial substitution of Pb^2+^ for Ag^+^ also contributes to the increased Mn^3+^ content in LPA and LEPA.

**Figure 6:**
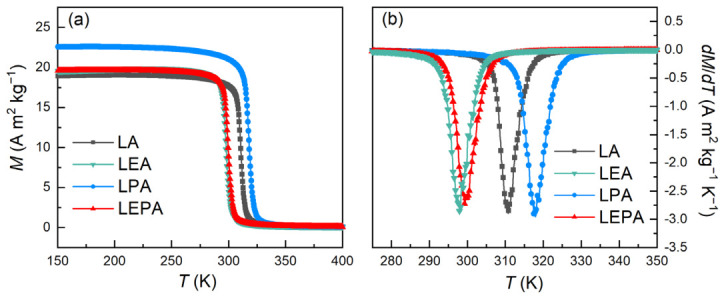
(**a**) ZFC *M*-*T* curves for the LA; LEA; LPA; and LEPA samples measured at 0.05 T; (**b**) Corresponding d*M*/d*T*-*T* curves.

Figure 6a reveals the *M*-*T* curves for the LA, LEA, LPA and LEPA manganites under the applied magnetic field of 0.05 T. All the samples exhibit typical ferromagnetic (FM)–paramagnetic (PM) transition behavior, with magnetization gradually decreasing as temperature increases. In this work, the *T*_C_ were determined by identifying the minima in the d*M*/d*T*-*T* curves, as shown in Figure 6b. The values of *T*_C_ for LA, LEA, LPA and LEPA are 310.8, 298.0, 318.0 and 299.3 K, respectively. Eu^3+^ doping alone leads to the increase in 〈$d_{\mathrm{Mn}\text{-}O}$〉 and the decrease in 〈$\theta_{\mathrm{Mn}\text{-}O\text{-}\mathrm{Mn}}$〉, resulting in the reduction of the bandwidth *W* and overlap between Mn_3d_ and O_2p_ orbitals, which consequently weakens the DE interaction and lowers the *T*_C_. In contrast, the partial substitution of Pb^2+^ for Ag^+^ induces the opposite changes in 〈$d_{\mathrm{Mn}\text{-}O}$〉 and 〈$\theta_{\mathrm{Mn}\text{-}O\text{-}\mathrm{Mn}}$〉 and alters the Mn^3+^/Mn^4+^ ratio, both of which contribute to enhancing the DE interaction and increasing the *T*_C_. Relative to single-doped samples, the *T*_C_ of LEPA lies between those of LEA and LPA, suggesting synergistic regulation of magnetic properties by Eu^3+^/Pb^2+^ co-doping.

**Figure 7:**
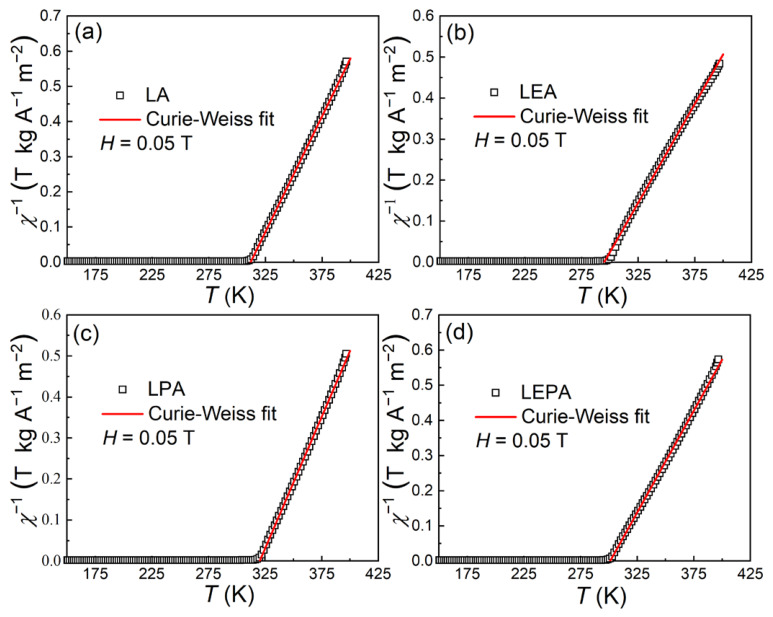
*χ*^−1^-*T* curves of (**a**) LA, (**b**) LEA, (**c**) LPA and (**d**) LEPA samples. The solid lines denote the Curie–Weiss law fitting results.

Generally, for ferromagnetic materials, the inverse magnetic susceptibility (*χ*^−1^) in the paramagnetic region follows the Curie–Weiss law [32]:

(6) $$\chi^{-1}\;=\;\frac{T\;-\theta_{P}}{C}$$

where *θ*_P_ and *C* represent the paramagnetic Curie–Weiss temperature and the Curie constant. Figure 7 illustrates the *χ*^−1^ vs. *T* curves for the present samples under a 0.05 T magnetic field. The red line depicts the linear fits to the Curie–Weiss law, and the derived *θ*_P_ values for LA, LEA, LPA and LEPA are 311.8, 294.8, 320.0 and 301.3 K, respectively. The experimental effective paramagnetic moment ($\mu_{\mathrm{eff}}^{\exp}$) can be calculated using the expression $C\;=\;N(\mu_{\mathrm{eff}}^{\exp}{\mu_{B})}^{2}/3k_{B}$, where *N* = *N*_A_/*M*_m_ is the number of magnetic ions per unit mass (*N*_A_ = 6.023 × 10^23^ mol^−1^ the Avogadro constant and *M*_m_ the molar mass), *k*_B_ = 1.38016 × 10^−16^ erg K^−1^ the Boltzmann constant and *μ*_B_ = 9.274 × 10^−21^ emu the Bohr magneton [33]. The theoretical effective paramagnetic moment is calculated as:

(7) $$\mu_{\mathrm{eff}}^{\mathrm{theo}}\;=\;\sqrt{x{\left[\mu_{\mathrm{eff}}\left({\mathrm{Eu}}^{3+}\right)\right]}^{2}\;+\;\left(0.6+y\right){\left[\mu_{\mathrm{eff}}^{\mathrm{theo}}\left({\mathrm{Mn}}^{3+}\right)\right]}^{2}\;+\;\left(0.4-y\right){\left[\mu_{\mathrm{eff}}^{\mathrm{theo}}\left({\mathrm{Mn}}^{4+}\right)\right]}^{2}}$$

here, *x* and *y* denote the nominal doping concentrations of Eu and Pb, respectively, with $\mu_{\mathrm{eff}}$(Eu^3+^) = 3.4 *μ*_B_ [34], $\mu_{\mathrm{eff}}^{\mathrm{theo}}$(Mn^3+^) = 4.9 *μ*_B_ and $\mu_{\mathrm{eff}}^{\mathrm{theo}}$(Mn^4+^) = 3.87 *μ*_B_ [35]. The $\mu_{\mathrm{eff}}^{\exp}$ values for the samples are 5.36, 6.27, 5.48 and 5.77 *μ*_B_, much higher than the $\mu_{\mathrm{eff}}^{\mathrm{theo}}$ values of 4.52, 4.54, 4.62 and 4.65 *μ*_B_. This discrepancy probably arises from the strong spin–orbit coupling in these samples, which drives the gyromagnetic factor above 2 [36].

**Figure 8:**
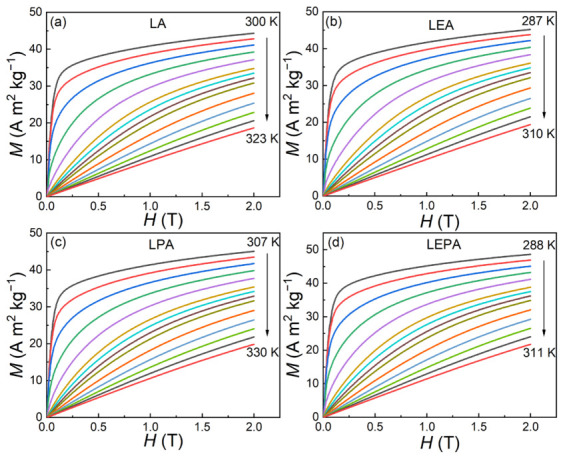
Isothermal magnetization curves of the (**a**) LA, (**b**) LEA, (**c**) LPA and (**d**) LEPA samples.

**Figure 9:**
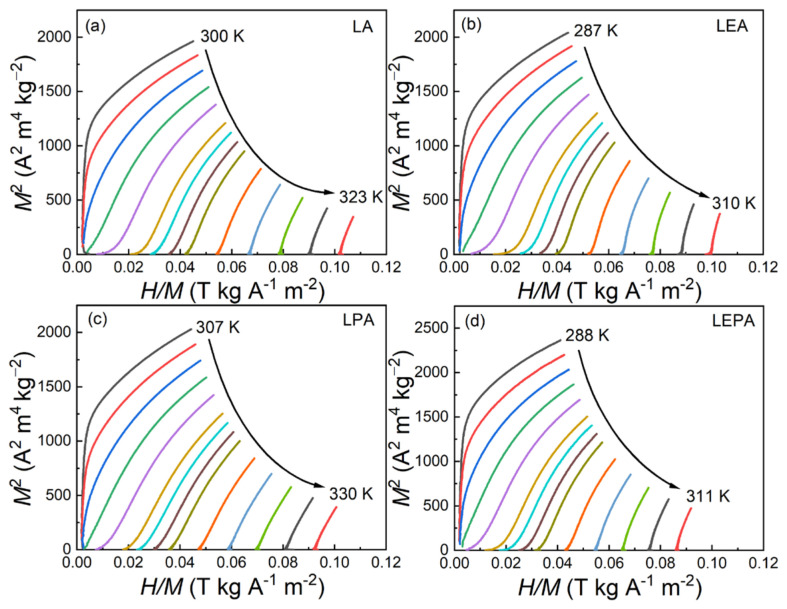
Arrott (*M*^2^-*H*/*M*) curves for the (**a**) LA, (**b**) LEA, (**c**) LPA and (**d**) LEPA samples.

Figure 8 displays the isothermal magnetization (*M*-*H*) curves for the LA, LEA, LPA, and LEPA samples measured near their *T*_C_ under the applied magnetic field of 0–2 T. At temperatures below *T*_C_, the magnetization increases rapidly with the magnetic field and tends to saturate, demonstrating typical ferromagnetic behavior. As the temperature rises, the magnetization gradually decreases, and the *M*-*H* curves become linear in the high-temperature region, indicating a transition from FM to PM states. To further characterize the nature of the magnetic phase transition, the corresponding Arrott plots (*M*^2^-*H*/*M*) are constructed from the *M*-*H* data, as shown in Figure 9. According to the Banerjee criterion [30], the positive slope of the Arrott plots for all the samples confirms the second-order FM-PM phase transitions. Compared with first-order magnetic phase transition materials, second-order counterparts exhibit negligible magnetic and thermal hysteresis, making them more favorable for practical magnetic refrigeration cycles, despite their smaller magnetic entropy changes [37].

In this work, the MCE of the samples is characterized by the isothermal magnetic entropy change |Δ*S*_M_|. Based on the thermodynamic Maxwell relation, the |Δ*S*_M_| can be expressed as [38]:

(8) $$\Delta S_{M}\left(T,\Delta H\right)\;=\;\int_{0}^{H_{\max}}\;{\left(\frac{\partial M}{\partial T}\right)}_{H}dH$$

**Figure 10:**
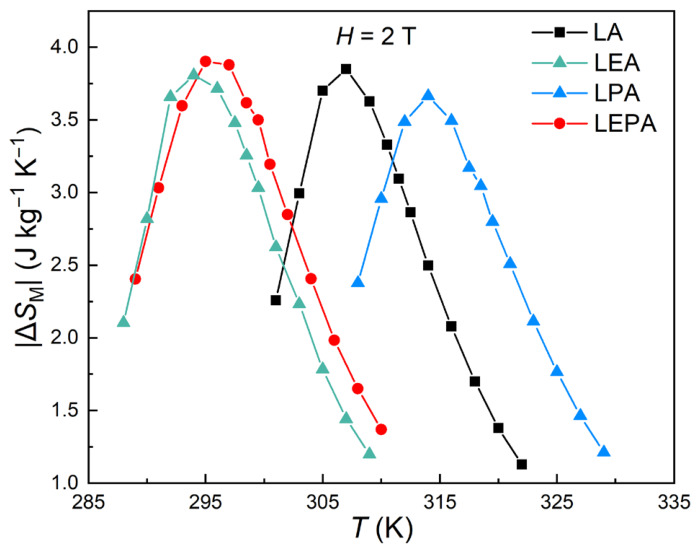
|Δ*S*_M_|-*T* curves measured for the LA; LEA; LPA; and LEPA samples under 2 T magnetic fields.

In practical measurements, for magnetization data obtained at discrete magnetic field and temperature intervals, Equation (8) can be approximated as [38]:

(9) $$\left|\Delta S_{M}\right|\;=\;\sum_{i}\;\frac{M_{i}-M_{i+1}}{T_{i+1}-T_{i}}\Delta H_{i}$$

where *M_i_* and *M_i_*_+1_ are the magnetization values at the adjacent temperatures *T_i_* and *T_i_*_+1_ within the corresponding magnetic field change intervals. Figure 10 presents the |Δ*S*_M_|-*T* curves of the four samples under a field change of 2 T. The maximum magnetic entropy change $\left|∆S_{M}^{\max}\right|$ is obtained in the vicinity of *T*_C_, with values of 3.85, 3.81, 3.66, and 3.90 J·kg^−1^·K^−1^ for LA, LEA, LPA, and LEPA, respectively. The MCE in perovskite manganites is usually affected by both the DE interaction and spin–lattice coupling [39]. Compared with the LA and LPA samples, the introduction of Eu^3+^ leads to a decrease in $\left|∆S_{M}^{\max}\right|$ for LEA and an increase for LEPA. Previous studies on the La_0.7−*x*_Eu*_x_*Sr_0.3_MnO_3_ and La_0.7−*x*_Eu*_x_*Ba_0.3_MnO_3_ systems [8,40] have shown that increasing Eu content enhances lattice distortion and weakens the DE interaction, resulting in a decrease in *T*_C_, while simultaneously strengthening the spin–lattice coupling, which in turn increases $\left|∆S_{M}^{\max}\right|$. Therefore, it is inferred that the introduction of Eu^3+^ in LEA and LEPA exerts similar effects. The slightly lower $\left|∆S_{M}^{\max}\right|$ of LEA compared to LA is more likely attributable to its smaller particle size, as reducing particle size generally suppresses both *T*_C_ and $\left|∆S_{M}^{\max}\right|$ [41]. As discussed above, for the LPA sample, Pb^2+^ doping enhances the DE interaction. Therefore, the lower $\left|∆S_{M}^{\max}\right|$ of LPA compared with LA may be attributed to weakened spin–lattice coupling [42]. Similar behavior has been reported in the La_0.78_Ca_0.22−*x*_Pb*_x_*MnO_3_ system, where *T*_C_ increases with Pb^2+^ doping, while $\left|∆S_{M}^{\max}\right|$ decreases [43].

Relative cooling power (*RCP*) is a key parameter for evaluating the overall performance of magnetocaloric materials, typically representing the amount of heat that can be transferred between the cold and hot reservoirs in an ideal refrigeration cycle. The equation for its calculation is as follows [38]:

(10) $$RCP\;=\;|\Delta S_{M}^{\max}|\;\times \;\delta T_{\mathrm{FWHM}}$$

where *δT*_FWHM_ is the full width at half maximum of the |Δ*S*_M_|-*T* curve. The *RCP* values for LA, LEA, LPA and LEPA are 64.65, 64.48, 68.24 and 73.42 J·kg^−1^, respectively, with LEPA possessing the highest value, indicating a combination of large |Δ*S*_M_| and good refrigeration performance.

For comparison, Table 4 lists the magnetocaloric parameters of selected magnetocaloric materials with *T*_C_ near room temperature. As shown, although the magnetocaloric performance of the present samples is inferior to that of Gd, it outperforms many La-based perovskite manganites with similar *T*_C_.

To examine the field-dependent scaling behavior of the magnetocaloric response, a universal curve model was employed [49]. In this approach, the |Δ*S*_M_| curves measured under different magnetic fields were rescaled using the reduced temperature *θ*:

(11) $$\theta \;=\;\left\{\begin{array}{c} -\frac{\left(T-T_{C}\right)}{\left(T_{1}-T_{C}\right)},\;T\;\leq \;T_{C}\\ \frac{\left(T-T_{C}\right)}{\left(T_{2}-T_{C}\right)},\;T\;>\;T_{C} \end{array}\right.$$

**Figure 11:**
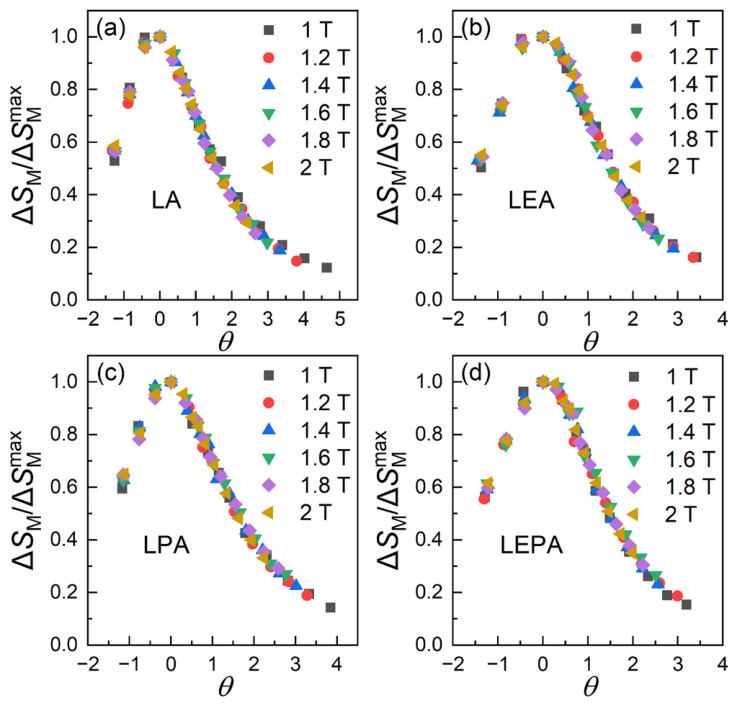
Universal curves of the (**a**) LA, (**b**) LEA, (**c**) LPA and (**d**) LEPA samples.

In this expression, *θ* denotes the rescaled temperature, while *T*_1_ and *T*_2_ satisfy Δ*S*_M_(*T*_1_)/Δ*S*_M_(max) = Δ*S*_M_(*T*_2_)/Δ*S*_M_(max) = 0.7. As presented in Figure 11, the normalized |Δ*S*_M_| curves of all samples collapse well onto a single master curve in the vicinity of *T*_C_. This behavior demonstrates good scaling consistency of the magnetocaloric response under different magnetic fields, confirming the field-dependent scaling behavior. This collapse, which is expected for a second-order transition, provides complementary support for the transition type indicated by the Arrott plots, in contrast to the scattered distribution characteristic of first-order transitions [50].

## 4. Conclusions

In this work, Eu^3+^ doping, Pb^2+^ doping, and Eu^3+^/Pb^2+^ co-doping were adopted to modulate microstructural, magnetic and magnetocaloric properties of La_0.80_Ag_0.20_MnO_3_-based manganites prepared by the Pechini sol–gel method. All samples retained a rhombohedral perovskite structure. Eu^3+^ doping resulted in much smaller particle size, indicating that the introduction of Eu alone suppresses crystallization ability, which can be avoided through Eu^3+^/Pb^2+^ co-doping.

Eu^3+^ and Pb^2+^ doping induce lattice contraction and expansion, respectively, and exert opposite effects on Mn-O bond lengths and Mn-O-Mn bond angles. Specifically, Eu^3+^ doping lengthens Mn-O bonds and decreases Mn-O-Mn bond angles, which weakens Mn_3d_-O_2p_ orbital overlap and the DE interaction, shifting *T*_C_ from 310.8 K to 298.0 K. In contrast, Pb^2+^ doping shortens Mn-O bonds and increases Mn-O-Mn bond angles, which enhances the orbital overlap and DE interaction, raising *T*_C_ to 318.0 K. Compared with single doping, Eu^3+^/Pb^2+^ co-doping exhibits a synergistic modulation effect, resulting in the *T*_C_ of 299.3 K for the LEPA sample.

All samples undergo second-order FM-PM phase transitions, with the magnetic entropy change peaks appearing near *T*_C_. Compared with LA, the decrease in $\left|∆S_{M}^{\max}\right|$ for LPA with Pb^2+^ doping may be attributed to weakened spin–lattice coupling, while the slight reduction for LEA with Eu^3+^ doping is likely due to its smaller particle size. Among the four samples, LEPA exhibits the highest $\left|∆S_{M}^{\max}\right|$ (3.90 J·kg^−1^·K^−1^) and the largest *RCP* under the 2 T field, which can be ascribed to the enhancement of spin–lattice coupling by Eu^3+^ outweighing the weakening effect of Pb^2+^.

In summary, this study demonstrates that rational multicomponent A-site design is an effective strategy for tailoring *T*_C_ and the magnetocaloric response in La-based perovskite manganites, providing a valuable basis for the compositional optimization of room-temperature magnetocaloric materials.

## Figures and Tables

**Table 1:** Refined structural parameters of the LA, LEA, LPA, and LEPA samples.

Compound	LA	LEA	LPA	LEPA
Space group	$R\bar{3}c$	$R\bar{3}c$	$R\bar{3}c$	$R\bar{3}c$
*a* (Å) = *b* (Å)	5.5072(5)	5.5064(1)	5.5125(9)	5.5102(2)
*c* (Å)	13.3438(7)	13.3400(9)	13.3599(9)	13.3410(5)
*V* (Å^3^)	350.49(6)	350.28(9)	351.59(9)	350.79(9)
〈$d_{\mathrm{Mn}\text{-}O}$〉 (Å)	1.960(3)	1.962(7)	1.956(0)	1.957(3)
〈$\theta_{\mathrm{Mn}\text{-}O\text{-}\mathrm{Mn}}$〉 (°)	163.5(2)	162.4(5)	164.5(3)	165.0(6)
*D*_SC_ (nm)	74.3	45.3	69.6	80.6
*D*_W-H_ (nm)	120.3	75.3	115.2	134.4
Microstrain (10^−4^)	3.74	5.73	3.59	3.64
〈*r*_A_〉 (Å)	1.3800	1.3773	1.3815	1.3775
〈*t*〉	0.9834	0.9824	0.9811	0.9797
*σ*^2^ × 10^3^	1.60	2.06	1.88	2.58
*W*	0.0938	0.0933	0.0947	0.0945
*R*_p_ (%)	3.20	2.71	3.07	3.09
*R*_wp_ (%)	4.16	3.55	4.01	3.94
*χ* ^2^	3.31	2.19	2.94	2.42

**Table 2:** Compositions of the LA; LEA; LPA; and LEPA samples obtained from EDX statistics.

Element		Content (at%) in Samples			
		LA	LEA	LPA	LEPA
La	Nominal	16	15.6	16	15.4
	Actual	16.46	15.16	16.13	14.14
Eu	Nominal	0	0.4	0	0.6
	Actual	0	0.38	0	0.49
Pb	Nominal	0	0	1	1
	Actual	0	0	0.98	0.94
Ag	Nominal	4	4	3	3
	Actual	2.52	2.50	2.03	2.20
Mn	Nominal	20	20	20	20
	Actual	18.51	19.37	18.45	17.16
O	Nominal	60	60	60	60
	Actual	62.51	62.59	62.41	65.07

**Table 3:** The fitting parameters of the Mn 2p peaks for LA, LEA, LPA, and LEPA samples.

Samples	Core Spectra	Mn States	Peak Position	Mn^3+^ Concentrate	Mn^3+^/Mn^4+^
LA	Mn 2p_3/2_	Mn^3+^	641.88	67.87%	2.11
		Mn^4+^	644.23		
	Mn 2p_1/2_	Mn^3+^	653.42		
		Mn^4+^	655.60		
LEA	Mn 2p_3/2_	Mn^3+^	641.89	68.87%	2.21
		Mn^4+^	643.87		
	Mn 2p_1/2_	Mn^3+^	653.44		
		Mn^4+^	655.14		
LPA	Mn 2p_3/2_	Mn^3+^	641.80	70.35%	2.37
		Mn^4+^	644.41		
	Mn 2p_1/2_	Mn^3+^	653.21		
		Mn^4+^	655.31		
LEPA	Mn 2p_3/2_	Mn^3+^	641.83	70.70%	2.41
		Mn^4+^	644.42		
	Mn 2p_1/2_	Mn^3+^	653.27		
		Mn^4+^	655.32		

**Table 4:** Magnetocaloric properties (*T*_C_, $\left|∆S_{M}^{\max}\right|$ and *RCP*) of selected magnetocaloric materials with *T*_C_ close to room temperature.

Samples	*T*_C_ (K)	*H* (T)	$\left|∆S_{M}^{\max}\right|$ (J·kg^−1^·K^−1^)	*RCP* (J·kg^−1^)	Ref.
LA	310.8	2	3.85	64.65	This work
LEA	298.0	2	3.81	64.48	This work
LPA	318.0	2	3.66	68.24	This work
LEPA	299.3	2	3.90	73.42	This work
La_0.80_Ag_0.20_MnO_3_	308	2	3.2	64	[12]
La_0.75_Sm_0.05_Sr_0.20_MnO_3_	310	2	1.80	74.30	[21]
La_0.78_Pb_0.22_MnO_3_	292	2	2.05	92.53	[44]
Pr_0.50_Sr_0.30_Ag_0.20_MnO_3_	305	2	1.34	58.2	[45]
La_0.75_Sm_0.05_Sr_0.20_MnO_3_	297	2	1.65	92	[46]
La_0.57_Nd_0.10_Sr_0.18_Ag_0.15_MnO_3_	310	2	2.97	75.33	[47]
Gd	292	2	5.20	226.90	[48]

## Data Availability

The original contributions presented in this study are included in the article. Further inquiries can be directed to the corresponding author.
